# Explaining the Female Preponderance in Adolescent Depression—A Four-Wave Cohort Study

**DOI:** 10.1007/s10802-023-01031-6

**Published:** 2023-02-04

**Authors:** Ida Sund Morken, Kristine Rensvik Viddal, Tilmann von Soest, Lars Wichstrøm

**Affiliations:** 1 Department of Psychology, NTNU – Norwegian University of Science and Technology, Trondheim, Norway; 2 Department of Psychology, University of Oslo, Oslo, Norway; 3 Department of Child and Adolescent Psychiatry, St. Olavs University Hospital, Trondheim, Norway

**Keywords:** Major depressive disorder (MDD), Dysthymia, Structural equation modelling, Negative life events, Sex difference

## Abstract

In the transition from childhood into adolescence, a female preponderance in depression emerges. Despite substantial empirical research to test theoretical propositions as to why this happens, our understanding is still limited. One explanation claims that girls become exposed to more stress (stress exposure model) whereas another proposes that girls become more vulnerable to the impact of stress (stress reactivity model) than boys when entering adolescence. Stressful life events (SLEs) and bullying victimization are established risk factors for adolescent depression. However, whether these factors contribute to the gender difference in depression is undetermined and thus investigated herein. Children (49.9% boys; *n* = 748) and parents from two birth cohorts in Trondheim, Norway, were followed biennially from ages 8 to 14 with clinical interviews about symptoms of depressive disorders and self-reports on SLEs. Teachers reported on bullying victimization. Prospective associations were investigated using an autoregressive latent trajectory model with structured residuals, examining within-person longitudinal associations while accounting for all time-invariant confounding effects. The number of depressive symptoms increased from ages 12 to 14 among girls. In the period before (ages 10 to 12), girls and boys were equally exposed to SLEs and bullying victimization. Increased stress (both SLEs and bullying victimization) at age 12 predicted increased depression at age 14 more strongly among girls than boys. Hence, increased impact—but not exposure—of SLEs and bullying victimization in girls may partly explain the emerging female preponderance in depression, in line with a stress reactivity model.

**Supplementary Information:**

The online version contains supplementary material available at 10.1007/s10802-023-01031-6.

Depression is a common disorder worldwide, is often recurrent, and is among the leading causes of years lived with disabilities (GBD 2019 Mental Health Collaborators, 2022). Throughout most of the lifespan, depressive symptoms and disorders occur more frequently among women than men, and this female preponderance emerges in early adolescence, by at least age 12 (Salk et al., 2017). Two of the stressors that repeatedly have been identified as risk factors for child and adolescent depression are stressful life events (SLEs) (Ge et al., 1994, 2001), and bullying victimization (Christina et al., 2021). However, whether these stressors are involved in explaining the emerging female preponderance in depression needs further inquiry—a task we undertake herein.

With profoundness of the sudden gender difference in depression as a backdrop, several etiological models have been developed to account for this phenomenon (Cyranowski et al., 2000; Hankin & Abramson, 2001; Hankin et al., 2007; Hyde & Mezulis, 2020; Hyde et al., 2008; Nolen-Hoeksema & Girgus, 1994). A common element of these models was first proposed by Nolen-Hoeksema and Girgus (1994), namely that gender differences in stress exposure might lead to a female preponderance in depression. This potential mechanism has been termed the *stress exposure model* (e.g., Hammen, 2009b; Hankin et al., 2007), and posits that when girls approach adolescence they experience more stressors than boys. Examples of such stressors are sexual harassment (e.g., Skoog et al., 2016) and relational problems with peers and friends (for a review, see Rose & Rudolph, 2006). Moreover, according to a stress-generation hypothesis (Hammen, 2009a), depression may lead to characteristics and behaviors that increase interpersonal stress, and this process could be more pronounced in adolescent girls than boys (Hammen, 2009a). Regardless of how girls become exposed to more stress than boys, increased levels of stress may partly explain why they also become more depressed. The *stress reactivity model* (Hammen, 2009b; Hankin et al., 2007), in contrast, suggests that girls become more vulnerable to stress than boys when entering adolescence. According to this model, adolescent girls may experience heightened negative emotional reactivity and hence a stronger impact of stress on depression, which, in turn, may partly explain why more girls than boys become depressed. Naturally, these two explanations are not mutually exclusive (e.g., Hyde et al., 2008). To examine stress exposure and reactivity as explanations for the gender difference in adolescent depression, we propose five criteria—three for stress exposure and two for stress reactivity—that need to be fulfilled. Importantly, strong tests of the exposure and reactivity models involving SLEs and bullying victimization according to these criteria are lacking.

## Stress Exposure Model

For the stress exposure model to be a valid explanation for the gender difference in depression, Criterion 1 states that girls should become exposed to more stress than boys just before the female preponderance in depression emerges (i.e., early adolescence), and not earlier (i.e., preadolescence). Previous research has found an increase in SLEs from childhood to adolescence (e.g., Larson & Ham, 1993). However, whether this increase is stronger for girls than boys is unclear. Even though a prior meta-analysis indicated that girls are exposed to more SLEs than boys, particularly during adolescence (Davis et al., 1999), later studies have not found gender differences in SLEs in adolescence (Jenness et al., 2019; Sund et al., 2003). Research on bullying victimization has also provided mixed results. Notably, most studies have investigated specific forms of bullying victimization, demonstrating, for example, that girls become more exposed to relational bullying and boys to physical bullying in mid-adolescence (Hager & Leadbeater, 2016), and that girls are more exposed to cyber victimization in early adolescence (Holfeld & Leadbeater, 2017). Other studies have not identified gender differences in the prevalence of relational bullying (Lepore & Kliewer, 2019) or cyber victimization (Díaz & Fite, 2019). However, given that all types of bullying victimization arguably thwart the fundamental need to belong, which in and by itself increases the risk for depression (Verhagen et al., 2018), we focus on bullying victimization in general. Studies on overall bullying victimization either portray an increase only among adolescent girls (Wendelborg, 2020), or find no such gender difference in prevalence (Sweeting et al., 2006). In sum, there is no consistent evidence indicating that girls become more exposed to SLEs or bullying victimization than boys just before the onset of the gender difference in depression.

Next, also pertaining to the stress exposure model, Criterion 2 states that increased stress in preadolescence should predict increased depression (at least among girls) in early adolescence, a prediction at the within-person level. Most prior research has utilized between-person information, asking whether those exposed to more SLEs and bullying victimization *than other adolescents* also become more depressed *than other adolescents*. However, other adolescents’ stress exposure and level of depression cannot be involved in the development of depression. As advocated by several developmentalists (Berry & Willoughby, 2017; Hamaker et al., 2020), traditional analytical approaches, such as ordinary cross-lagged analyses of longitudinal data, do not disentangle within- from between-person information and therefore provide limited information from which to draw causal inferences (Berry & Willoughby, 2017). Importantly, the results from studies using between-person information can be influenced by time-invariant confounding effects, such as stable effects of genetics increasing the risk of both SLEs and depression (Clarke et al., 2018) or a persistent harsh parenting style increasing the risk for both bullying victimization and depression (Tang et al., 2018). Thus, to more closely approximate questions of causality (see e.g., Lervåg, 2020) while examining Criterion 2, we need to obtain information about the within-person association between preadolescent stress and prospective increased depression.

Finally, to support stress exposure as an explanation for the female preponderance in depression, Criterion 3 states that the sudden gender difference in depression should be accounted for (i.e., mediated) by an increasing gender disparity in levels of stress. However, at present, we do not know whether SLEs or bullying victimization mediate the gender difference in depression.

## Stress Reactivity Model

To support a stress reactivity model, two criteria must be met. Criterion 4 states that increased stress should be more strongly associated with increased depression in girls than boys—and at the within-person level. Moreover, Criterion 5 states that this gender difference in stress reactivity should first appear in late childhood or early adolescence. Some regression-type research indicates that SLEs predict the level of depression to a stronger degree in girls than in boys during early adolescence (Ge et al., 1994), but whether this is the case for bullying victimization is unclear (Christina et al., 2021; Lepore & Kliewer, 2019). These findings notwithstanding, we lack research on whether associations are stronger among girls at the within-person level (Criterion 4) and whether they are specific to early adolescence as opposed to middle childhood (Criterion 5).

## The Current Study

We investigate whether and how SLEs and bullying victimization contribute to explaining the emerging female preponderance in depression. We do this through biennial follow-ups of a community sample spanning from middle childhood to adolescence and by measuring depressive symptoms as defined by the Diagnostic and Statistical Manual of Mental Disorders (5th ed.; *DSM-5*; American Psychiatric Association, 2013). We examine whether the female preponderance in depression is partially explained by (i) increased stress exposure, where girls become more exposed to SLEs and bullying victimization than boys before this gender gap emerges, or (ii) increased stress reactivity, where SLEs and bullying victimization predict depressive symptoms to a stronger degree among girls than boys when entering adolescence. We hypothesize that both the stress exposure and reactivity explanations partly account for the emerging gender difference in depression. These explanations are examined adhering to the abovementioned five criteria.

## Methods

### Participants and Procedure

The Trondheim Early Secure Study (TESS) (Steinsbekk & Wichstrøm, 2018) comprises children from the 2003 and 2004 birth cohorts in Trondheim, Norway (*N* = 3,456). A letter of invitation along with the Strengths and Difficulties Questionnaire (SDQ) version 4–16 (Goodman et al., 2000) was sent to the children’s homes prior to the age 4 routine health check-up. Almost all children met with their parents at the check-up (*n* = 3,358). Parents received information about the study orally and in writing from the health nurse and written consent was obtained. Study procedures were approved by the Regional Committee for Medical and Health Research Ethics, Mid-Norway (approval number 2009/994).

To increase statistical power, children were divided into four strata based on their SDQ score (0–4, 5–8, 9–11, 12–40), and the probability of being selected increased with increasing scores (37%, 48%, 70%, and 89% from the respective strata). This oversampling of mental health problems was accounted for in the analyses. The drop-out rate after consent at the well-child clinic did not differ across the four SDQ strata; χ^2^(3) = 5.70, *p* = 0.127, or by gender; χ^2^(3) = 0.23, *p* = 0.973. Of the 1,250 children randomly selected for the study, 1,007 were successfully enrolled at Time 1 (*M*_*age*_ = 4.59, *SD* = 0.25; 49.1% boys) (for a flowchart, see Figure S1). Testing occurred biennially. Given that our research questions pertain to explaining depression in the transition from middle childhood to early adolescence, we included data from ages 8 (T3: *M*_*age*_ = 8.79, *SD* = 0.23), 10 (T4: *M*_*age*_ = 10.51, *SD* = 0.17), 12 (T5: *M*_*age*_ = 12.50, *SD* = 0.14) and 14 years (T6: *M*_*age*_ = 14.35, *SD* = 0.14). Attrition rates between waves of data collection were as follows: T3-T4: 0.14%, T4-T5: 5.26% and T5-T6: 4.51% (for more details, see Figure S1). Participants with information from at least one data wave composed the analytical sample (*n* = 748). Overall, attrition was unrelated to the study variables, including symptoms of Major Depressive Disorder (MDD) and dysthymia, and SLEs and bullying victimization measured at ages 4 and 6 years. However, more symptoms of MDD (OR = 1.39, 95% CI [1.15, 1.70]) and dysthymia (OR = 1.35, 95% CI [1.12, 1.64]) at age 12 predicted attrition at age 14, and more bullying victimization at age 6 predicted attrition at ages 10, 12 and 14 (all ORs = 1.02, 95% CI [1.01, 1.03]). The sample characteristics are presented in Table 1. Even though the above analyses suggested that attrition was selective according to study variables, they should be interpreted in the context of the number of attrition analyses conducted. An overall test, the Little Missing Completely at Random (MCAR) test (Little, 1988) was therefore conducted. The results showed that data were just barely shy of being MCAR, Δχ^2^(220) = 256.01, *p* = 0.048, whereas the normed Little’s test was 1.16 (normed value < 2 suggesting missing at random–MAR) thus indicating that data were at least MAR.

**Table 1:** Sample characteristics

Characteristics	%
Gender of child	
Male	48.9
Female	51.1
Gender of parent informant	
Male	16.7
Female	83.3
Parent informant	
Biological parent	98.3
Adoptive parent	1.3
Foster parent	0.4
Biological parents’ marital status	
Married	59.3
Cohabitating > 6 months	21.9
Cohabitating < 6 months	0.4
Divorced/separated/no longer cohabitating	16.4
Widowed	0.1
Never lived together	1.9
Ethnic origin of biological mother	
Norwegian	93.0
Western Countries	2.7
Other Countries	4.3
Ethnic origin of biological father	
Norwegian	91.0
Western Countries	5.8
Other Countries	3.2
Informant parents’ socioeconomic status	
Leader	17.5
Professional, higher level	30.1
Professional, lower level	30.1
Formally skilled worker	18.5
Farmer/fishermen	0.2
Unskilled worker	3.6

### Measures

Depressive symptoms were measured as symptoms of MDD and dysthymia according to DSM-5 criteria (American Psychiatric Association, 2013) using a semi-structured psychiatric interview, the Child and Adolescent Psychiatric Assessment (CAPA) (Angold & Costello, 2000). Children and parents were interviewed separately. A symptom was considered present if reported as occurring in the three months prior by either respondent. Inter-rater reliabilities among blinded coders of 15% of audiotapes of CAPA interviews were ICC = 0.87 for symptoms of MDD and ICC = 0.85 for symptoms of dysthymia. A symptom count score was created as the sum of MDD and dysthymia symptoms.

SLEs were measured by parent and child reports on 31 SLEs occurring since the last visit (two years), ranging from important life events (e.g., new sibling, parents separated or divorced) to very serious ones (e.g., sexual abuse) (see Appendix S1 for a complete list). A SLE was considered present if reported by either respondent, and a SLE total score was created as the sum of the number of SLEs. Given the wide range of seriousness in these events, we tested the possibility that any association between depression and life-events was driven by more serious or less serious events by comparing the correlations between depression and important life events to the correlations to events with a substantial potential for grave physical and mental harm, using the Satorra-Bentler scaled chi-square difference test (Satorra & Bentler, 2001). Allowing these correlations to be different did not improve model fit as compared to the correlations being identical, Δχ^2^(4) = 2.49, *p* = 0.952, a fact suggesting that they did not differ and that the depression-SLE association was not different according to the seriousness of the SLEs.

Bullying victimization was measured by a teacher version of the Olweus Bully Victim Questionnaire (OBVQ) (Solberg & Olweus, 2003), completed by the participant’s primary teacher. This teacher version of the OBVQ consists of five items pertaining to both physical bullying and social exclusion (α = 0.69 to 0.79) tapping the frequency of physical harm, verbal abuse, social exclusion, been overlooked, and belongings hidden or destroyed, during the last 3 months. Response options ranges from *Never*, *Rarely*, *1–3 times per month*, *1–4 times per week* to *Everyday*.

Sociodemographic information on child and parent was reported by the parent during the diagnostic interview. Gender was coded (0 = boy, 1 = girl) based on the child’s national identification number, in which the child’s biological sex at birth is registered.

### Statistical Analyses

As we did expect a change in the overall level of depression, and potentially also in SLEs and bullying victimization we employed autoregressive latent trajectory models with structured residuals (ALT-SR) (Berry & Willoughby, 2017) because they can accommodate linear and non-linear changes over time. Changes from one time point to the next was captured by latent change scores. In line with Orth et al.’s (2022) tentative suggestions, we considered cross-lagged associations with standardized regression coefficients of 0.03, 0.07, and 0.12 to indicate small, medium, and large effect sizes, respectively.

As our goal was to explain the female preponderance in depression, we focused on the age span when this gender difference is first expected to emerge—in early adolescence (i.e., ages 12 to 14) (Salk et al., 2017). To examine whether changes were specific to this age period we also analyzed the two age spans just prior to it (ages 8 to 10 and 10 to 12). Imbedded in the explanatory stress-exposure and stress-reactivity models is a causal relation between stress (e.g., bullying victimization) and depression. Hence, the increase in the exposure (stress) should occur before the increase in the outcome (depression). The possibility of a stress generating effect of depression (Hammen, 2009a) on SLEs and bullying victimization should be taken into account. Hence, a parallel increase in stress from ages 12 to 14 would not suffice as unequivocal predictor of change in depression from 12 to 14, because increased stress could be an effect of increased depression in the same period, not a predictor of it. Provided we find the expected increase in depression from ages 12 to 14, the increase in stress should therefore occur in period before, that is from ages 10 to 12.

**Fig. 1:**
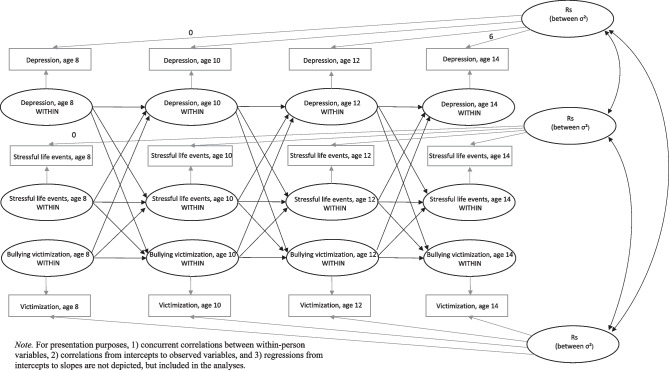
Theoretical autoregressive latent trajectory model with structural residuals model of depression, stressful life events and bullying victimization

The three criteria pertaining to the stress exposure model were tested in the following way: Criterion 1, whether girls become more exposed to stress than boys just prior to the emergence of a gender difference in depressive symptoms (i.e., ages 10 to 12) and not before (i.e., ages 8 to 10), was examined by inspecting whether the latent change in SLEs and bullying victimization increased among girls, specifically from ages 10 to 12 and not 8 to 10. Second, we examined whether any increases in SLEs or bullying victimization from ages 10 to 12 were predicted by female gender. Criterion 2, whether increased SLEs and bullying victimization predicted later depressive symptoms in girls at the within-person level, was tested by applying a modified version of the ALT-SR model (Berry & Willoughby, 2017) depicted in Fig. 1. In this model, between-person differences in depressive symptoms, SLEs and bullying victimization were captured by the intercept (representing the mean level) and slope (representing growth) in each of these three constructs, while within-person scores at each timepoint provide information about a person’s deviation from his or her intercept and slope. In the traditional ALT-SR, the slope is set to be linear across all timepoints. Because the development of depressive symptoms, SLEs and bullying victimization are not necessarily expected to follow a linear pattern, we applied a latent basis model where the growth was freely estimated from the data, anchoring the slopes at ages 8 and 14. Criterion 3, whether the gender difference in depressive symptoms is explained by a potential increase in the study stressors, was examined by mediation analyses using Sobel’s test (Mplus does not enable bootstrapping with population weights).

Regarding the stress reactivity explanation, Criterion 4, whether SLEs and bullying victimization predicted depressive symptoms more strongly for girls than boys at the within-person level, was tested by adding an interaction term between gender and SLEs and bullying victimization, respectively, at ages 10 and 12 in the ALT-SR models following procedures described by Mulder and Hamaker (2020). Finally, Criterion 5, whether a potentially stronger association for girls than boys was specific to early adolescence, was examined by inspecting whether the gender differences in the within-person associations between SLEs/bullying victimization and depressive symptoms were present only from ages 12 to 14 and not from ages 10 to 12.

All analyses were performed in Mplus 8.5 using a robust maximum likelihood estimator and probability weights to correct for the oversampling of children with mental health problems. Missing data were handled using a full information maximum likelihood (FIML) procedure under the assumption that data was MAR.

## Results

**Fig. 2:**
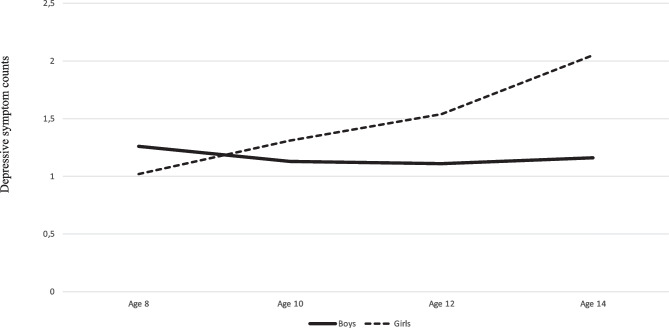
Development of depressive symptom counts from age 8 to age 14 for boys and girls

The results showed rather low counts of depressive symptoms at ages 8 to 12, with scores between 1.0 and 1.5 for both genders, but with a sudden increase for 14-year-old girls to 2.1, while boys’ depressive symptoms count remained at a stable low level (see Table 2 and Fig. 2). This increase was mirrored by female gender being associated with depressive symptoms at age 14 (*r* = 0.16, 95% CI [0.05, 0.24]) but not at ages 12 (*r* = 0.08, 95% CI [-0.03, 0.17]) or 10 (*r* = 0.02, 95% CI [-0.07, 0.10]). At age 8, female gender was associated with fewer symptoms (*r* = -0.13, 95% CI [-0.23, -0.05]). Latent change scores analyses confirmed the emerging female preponderance in depression in early adolescence, as female gender predicted an increased number of depressive symptoms from ages 12 to 14 but not from ages 10 to 12 or 8 to 10 (see Table 2). Correlations between study variables are provided in Table S1.

**Table 2:** Gender differences in stressful life events (SLEs) and bullying victimization, and mean level change, ages 8–14

	Boys		Girls		Gender differences in latent change scores
	Mean level (SD)	*p*-value of 2-year change	Mean level (SD)	*p*-value of 2-year change	B [95% CI]
Depression					
Age 8	1.26 (1.77)		1.02 (1.66)	-	-
Age 10	1.13 (1.65)	0.176	1.31 (1.79)	0.053	0.14 [-0.02, 0.29]
Age 12	1.11 (1.96)	0.874	1.54 (2.04)	0.214	0.17 [-0.02, 0.35]
Age 14	1.16 (2.09)	0.061	2.05 (2.89)	0.003	0.23 [0.01, 0.44]
Stressful life events					
Age 8	1.10 (1.35)		0.96 (1.33)	-	-
Age 10	1.14 (1.35)	0.724	1.17 (1.51)	0.038	0.03 [-0.07, 0.13]
Age 12	1.63 (1.81)	< 0.001	1.63 (1.67)	< 0.001	-0.00 [-0.12, 0.11]
Age 14	1.94 (1.66)	0.047	2.20 (1.79)	< 0.001	0.11 [-0.01, 0.23]
Bullying victimization					
Age 8	1.08 (1.04)		1.03 (1.10)		
Age 10	1.02 (1.08)	0.349	0.89 (0.96)	0.175	-0.04 [-0.11, 0.03]
Age 12	0.99 (1.41)	0.854	0.85 (0.98)	0.954	-0.04 [-0.12, 0.04]
Age 14	0.60 (0.90)	0.001	0.66 (0.90)	0.002	0.03 [-0.04, 0.09]

### Stress Exposure Model

**Fig. 3:**
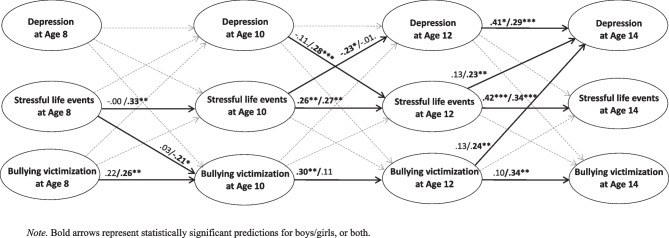
Within-person standardized regression coefficients from autoregressive latent trajectory model with structured residuals for boys/girls

To examine the stress exposure explanation, we first tested Criterion 1—girls becoming more exposed to stress than boys in the period prior to the female preponderance in depressive symptoms. In girls, SLEs significantly increased from ages 8 to 10 and 10 to 12, while bullying victimization remained stable (see Table 2). However, girls did not become more exposed to either SLEs or bullying victimization than boys in any of these age spans (see Table 2). Thus, Criterion 1 was not fulfilled. Regarding Criterion 2—stress predicting an increased number of depressive symptoms in girls at the within-person level, girls’ depressive symptoms at age 14 were predicted by SLEs (B = 0.39, *SE* = 0.12, 95% CI [0.15, 0.62]) and bullying victimization (B = 0.67, *SE* = 0.25, 95% CI [0.18, 1.15]) at age 12 in ALT-SR analyses. Standardized regression coefficients of the associations are presented in Fig. 3 and indicate large effect sizes between the associations of both SLEs and bullying with girls’ depressive symptoms at age 14 (i.e., β ≥ 0.12). However, when examining Criterion 3—the gender effect on depressive symptoms at age 14 being mediated by increased SLEs or bullying victimization–at age 12, the results revealed no such effects (B = -0.01, *SE* = 0.03, 95% CI [-0.08, 0.05] and B = -0.04, *SE* = 0.04, 95% CI [-0.12, 0.03], respectively). In sum, Criteria 1 and 3 for increased stress exposure were not met.

### Stress Reactivity Model

When examining the stress reactivity model, Criterion 4—stress predicting depressive symptoms more strongly in girls than boys at the within-person level—SLEs at age 12 predicted depressive symptoms at age 14 among girls (B = 0.39, *SE* = 0.12, 95% CI [0.15, 0.62]) but not among boys (B = 0.16, *SE* = 0.12, 95% CI [-0.08, 0.40]) (see Fig. 3 for standardized values), and a significant gender*SLEs interaction (B = 0.21, *SE* = 0.10, 95% CI [0.02, 0.39]) was detected. A similar pattern was observed for bullying victimization, which predicted depressive symptoms in girls (B = 0.67, *SE* = 0.25, 95% CI [0.18, 1.15]) but not boys (B = 0.25, *SE* = 0.21, 95% CI [-0.16, 0.66]) (see Fig. 3 for standardized values), with a significant gender*bullying victimization interaction (B = 0.53, *SE* = 0.20, 95% CI [0.15, 0.92]). Finally, Criterion 5 states that this gender difference should not be present before the emerging female preponderance in depression. As seen in Fig. 3, no such predictive effects were detected at younger ages, a finding supported by a nonsignificant gender*SLEs interaction (B = -0.06, *SE* = 0.06, 95% CI [-0.18, 0.06]) and gender*bullying victimization interaction (B = 0.01, *SE* = 0.14, 95% CI [-0.27, 0.29]) from ages 10 to 12. Thus, both criteria pertaining to the stress reactivity explanation were met.

## Discussion

The female preponderance in depression first emerges in early adolescence, and adolescent SLEs and bullying victimization are consistent predictors of depression. However, it is undetermined whether SLEs and bullying victimization can explain why the gender difference in depression emerges at this point in development. We examined two psychosocial explanations—a stress exposure model (girls becoming more exposed to stress than boys) and a stress reactivity model (early adolescent girls reacting to stressors with more depression than boys) by systematically testing whether specific criteria were fulfilled. We examined these criteria by drawing on a representative birth cohort sample followed biennially from age 8 to 14 years and applying ALT-SR methodology to illuminate within-person development. Our results showed the expected female preponderance in depression: the number of DSM-5 defined symptoms of major depression and dysthymia increased sharply from ages 12 to 14 among girls but not among boys. Furthermore, a stress exposure explanation was not supported, whereas a stress reactivity explanation was supported; girls who were exposed to more SLEs and bullying victimization at age 12 developed an increased number of depressive symptoms at age 14, with standardized regression coefficients indicating large effect sizes. No such associations were seen among boys or at earlier timepoints. Notably, the five criteria proposed herein may be applied in future studies on other stressors’ role in explaining the female preponderance in depression.

Although the present study was not positioned to unravel the underlying mechanisms for the assumed stress reactivity, we draw attention to some possibilities. First, puberty likely plays a role in stress regulation. Gonadal hormone secretion increases in puberty, which, in turn, is associated with gender differences in the hypothalamic–pituitary–adrenal axis response to stress, including cortisol production (Heck & Handa, 2019). Of current interest, there is evidence that adolescent girls evince lower cortisol levels when exposed to social stress than boys (Bouma et al., 2009), which may increase their vulnerability to developing depression (Colich et al., 2015). Arguably, therefore, the altered stress regulation in puberty may reinforce the negative effects of SLEs and bullying victimization in girls in particular. Another potential mechanism involves adolescent girls’ use of maladaptive cognitive coping strategies when faced with stress, such as rumination, which increases the risk for depression (Aldao et al., 2010). Indeed, girls tend to ruminate more than boys and more so in early adolescence than in late childhood (Hampel & Petermann, 2005), making them more vulnerable to depressive reactions to stress in this particular developmental period. To clarify the practical implications of the present findings, future studies should delineate the mechanisms involved in girls’ increased reactivity to SLEs and bullying victimization. In turn, preventive and treatment efforts may target the most potent mechanisms.

Our study found that girls did not become more exposed to either SLEs or bullying victimization than boys from age 10 to age 12, and these stressors did not mediate the gender effect on depressive symptoms. In effect, the increased stress exposure model was not supported. Previous studies have reported mixed evidence for gender differences in the prevalence of or increase in SLEs and bullying victimization in early adolescence, and the current findings coincide with those reporting no gender difference in SLEs (Jenness et al., 2019; Sund et al., 2003) or overall bullying victimization (Sweeting et al., 2006) in early adolescence. The discrepancies between findings may be attributed to a range of methodological and sample differences, including differences in the specific SLEs studied, age of participants, secular period, populations, and nationalities. For example, Hankin et al. (2007) found in a sample of US adolescents that girls were exposed to more interpersonal stress than the boys were, whereas Sund et al. (2003) found that Norwegian adolescent girls and boys were exposed to a similar amount of interpersonal stress.

We focused on two stressors that are established risk factors for depression: SLEs and bullying victimization. Whether other relevant stressors, such as daily hassles (Hankin et al., 2007) and peer sexual harassment victimization (Dahlqvist et al., 2016), follow exposure or reactivity patterns awaits future research. Importantly, to provide strong tests of exposure and reactivity explanations, such inquiries should cover the whole age-span from late childhood (i.e., even *before* the gender difference in depression appears) until adolescence. In a related vein, SLEs and victimization may have different effects on maintaining or widening the gender difference in depression in later adolescence (Salk et al., 2017), and other contributing factors may differ between these developmental periods. Our results are therefore specific to the development of depressive symptoms in the early adolescent period. Finally, the present results are specific to symptoms of depression and do not preclude the possibility of boys reacting more strongly than girls with symptoms of other disorders.

At age 8, boys evinced slightly more depressive symptoms than girls. As pointed out in previous research (reviewed by e.g., Salk et al., 2017), there are some reports of a male preponderance in depression in early childhood, whereas others do not find this difference. Hence, whether there is a gender difference in early childhood awaits further inquiry.

### Limitations

While this study has a range of strengths, including a representative community sample, clinical interviews to assess depressive symptoms, multiple informants, repeated assessments before and through the crucial years when the gender differences emerge, and a solid statistical approach to assess predictions at the within-person level, we acknowledge several limitations. First, children with more depressive symptoms at age 12 more often dropped out of the study by age 14, potentially resulting in underestimating the increase in depressive symptoms during this period. However, considering that our prime interest was gender differences in prevalence and associations and that we applied an FIML approach to missingness, selective attrition likely did not have a major impact on the results. Second, although we adjusted for all time-invariant confounders, time-varying factors, such as bodily changes associated with puberty or increased risk behavior in adolescence, may still have influenced both stress and depressive symptoms. Third, we studied symptom counts. Although there is no compelling evidence pointing to depression being categorical in nature (Haslam et al., 2012), we cannot be sure that our findings apply to depressive disorders. Fourth, we captured depressive symptoms occurring in the prior 3-month period, and symptoms occurring between our 2-year intervals of observation might have been missed. Fifth, we summed the number of SLEs, which differed considerably in frequency and seriousness; thus, we were not able to discern the effect of specific SLEs. Sixth, we assessed bullying victimization based on teacher reports. As such, bullying victimization that occurs outside of the school context, perhaps most notably cyber victimization (Díaz & Fite, 2019), might not have been captured. Even though those who are victimized online are often victimized at school as well (Wendelborg, 2020), the rate of overall bullying victimization might have been deflated. Seventh, gender was measured as biological sex assigned by birth (either girl or boy), thus not taking gender identity into account. Mounting evidence suggests that non-binary youth are at increased risk for psychiatric symptoms (e.g., Johansson et al., 2022; Price-Feeney et al., 2020) and theories on the gender difference in depression are limited by the gender binary. Notably, current surveys in Norway indicate that 0.4% of adults do not consider themselves as males or females and that 0.005% do not know (Statistics Norway, 2021). Although these numbers likely are higher among youths, the models that were tested herein would demand larger sample size than ours. Finally, future studies should include direct assessments of stress reactivity (e.g., behavioral observation or electrophysiological or hormonal measures).

### Conclusions

The current study is the first to examine and present support for the notion that increased reactivity to both SLEs and bullying victimization in early adolescent girls may contribute to explaining the emerging female preponderance in depression. These findings highlight the transition to early adolescence as critical for preventive interventions. Professionals implementing such efforts should take into account that exposure to SLEs and bulling victimization, occurring already in preadolescence, might confer a heightened risk for depressive symptoms for early adolescent girls in particular.

## Supplementary Information

Below is the link to the electronic supplementary material.

Supplementary file1 (PDF 153 KB)

## Data Availability

Due to conditions for consent from participants, data cannot be shared.
