# Supplementary material for: Explaining the Female Preponderance in Adolescent Depression—A Four-Wave Cohort Study
Source: Res Child Adolesc Psychopathol. 2023 Feb 4;51(6):859–69. doi: 10.1007/s10802-023-01031-6 (PMC10195739; doi:10.1007/s10802-023-01031-6)
Supplement: Supplementary file 1 — Supplementary file1 (PDF 153 KB) [file 10802_2023_1031_MOESM1_ESM.pdf]

Figure S1

Flow chart of recruitment and follow-up

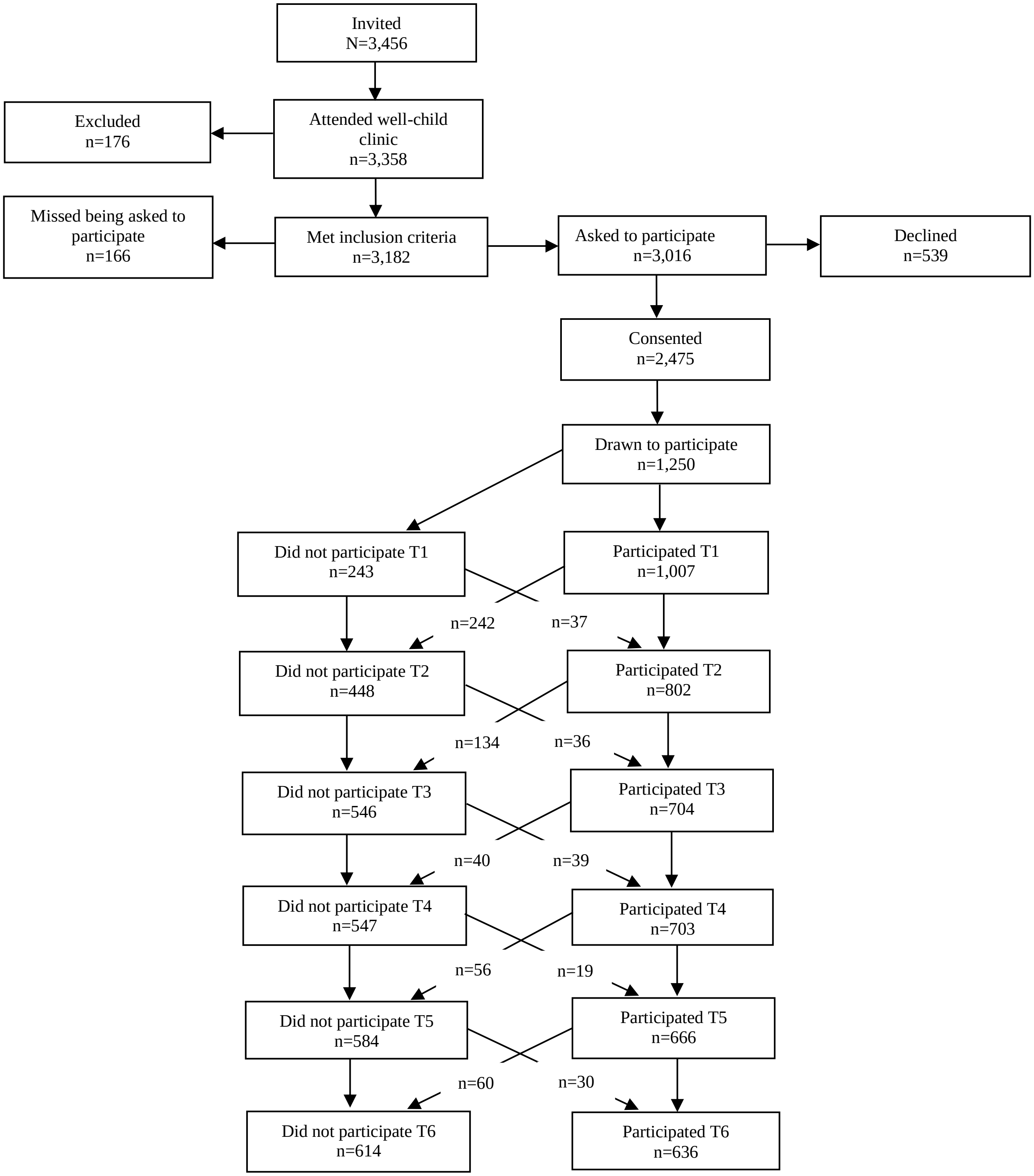

Note. Number of participants at the various assessment points is based on the number of participants invited to participate (*n* = 1,250) minus those who did not participate at the respective measurement point (i.e., T1, T2).

Appendix S1

List of stressful life events

1. *Have one or more children that are not your siblings come to live in your home?*
2. *Have you gotten new half- or full siblings?*
3. *Have your parents separated or divorced and no longer live together?*
4. *Did a new adult move into your home, e.g., a new girl/boy-friend or partner to your parents?*
5. *Did such a new adult move out of your home, e.g., a new girl/boy-friend or partner?*
6. *Have you moved to a new place?*
7. *Have you changed your school?*
8. *Have your parents been evaluated at or admitted to a hospital for more than 24 hours?*
9. *Have either of your parents suffered a life-threatening illness which made them unable to work?*
10. *Have either of your parents been admitted to a hospital for more than 7 days?*
11. *Have you lost contact with someone important to you (such as a friend or relative)?*
12. *Have you and a best friend parted because you had a quarrel or fight (seems to be permanent)?*
13. *Have you and a girl/boy-friend broken up because you fought, were no longer in love, or because one of you moved?*
14. *Did a pet you felt close to die?*
15. *Have either of your parents been in prison?*
16. *Has your family's income been substantially less than usual?*
17. *Have you been forced to live elsewhere than your home for a period?*
18. *Have you ever gotten very sick (risk of death or a serious illness)?*
19. *Have you ever been admitted to a hospital for more than 24 hours?*
20. *Have you ever been in a serious accident? (fire/burn, poisoning, traffic-accident, near drowning, bitten by an animal, ect.?)*
21. *Has anyone close to you died (parents or full- half- and step- siblings)?*
22. *Have you ever experienced a serious natural disaster, such as a storm, hurricane, flood, earthquake?*
23. *Have you ever been in a terrible fire?*
24. *Have you ever seen or heard something horrible happen to anyone?*
25. *Have you ever heard about something horrible happen to someone you care about, but not seen it yourself?*
26. *Have you ever had contact with something that could make you very sick or die?*
27. *Have you or someone you were with ever hurt another person seriously?*
28. *Have you ever been hit or hurt by somebody?*
29. *Have anyone in your family ever hit or hurt you badly?*
30. *Have you ever been kidnapped or locked up against your will?*
31. *Have you ever been exposed to sexual abuse?*

Note. Parents and children were asked to affirm if the following events occurred during the last two years.

Table S1

Associations between depression, stressful life events, and bullying victimization, ages 8-14

	1	2	3	4	5	6	7	8	9	10	11	12	13
1. Gender	1												
2. Depression – age 8	-.11*	1											
3. Depression – age 10	.02	.31***	1										
4. Depression – age 12	.09*	.37***	.38***	1									
5. Depression – age 14	.14**	.26***	.36***	.55***	1								
6. Stressful life events – age 8	-.05	.05	-.01	.08	.10	1							
7. Stressful life events – age 10	.02	-.00	.08	.01	.12**	.38***	1						
8. Stressful life events – age 12	-.06	.02	.18**	.12**	.23***	.29***	.39***	1					
9. Stressful life events – age 14	.07	.06	.11*	.13*	.22***	.18**	.18**	.45***	1				
10. Bullying victimization – age 8	-.00	.21***	.04	.14*	.18**	.11	.08	.11*	.12	1			
11. Bullying victimization – age 10	-.05*	.19***	.09	.12*	.15**	-.02	-.03	.03	.09	.39***	1		
12. Bullying victimization – age 12	-.11*	.09	.08	.21***	.25***	.02	.03	-.02	.12*	.22***	.33***	1	
13. Bullying victimization – age 14	.03	.11	.07	.12*	.20**	.02	.02	.01	.12*	.21***	.23**	.39***	1

r = Pearson product-number coefficient. * indicates *p* <.05, ** indicates *p* <.01, *** indicates *p* <.001.
